# Selection of endogenous control and identification of significant microRNA deregulations in cervical cancer

**DOI:** 10.3389/fonc.2023.1143691

**Published:** 2023-04-24

**Authors:** T. Stverakova, I. Baranova, P. Mikyskova, B. Gajdosova, H. Vosmikova, J. Laco, V. Palicka, H. Parova

**Affiliations:** ^1^ Department of Clinical Biochemistry and Diagnostics, Charles University, Faculty of Medicine in Hradec Králové and University Hospital Hradec Králové, Hradec Králové, Czechia; ^2^ Biomedical Center Martin, Jessenius Faculty of Medicine in Martin, Comenius University in Bratislava, Martin, Slovakia; ^3^ The Fingerland Department of Pathology, Charles University, Faculty of Medicine in Hradec Králové and University Hospital Hradec Králové, Hradec Králové, Czechia

**Keywords:** cervical cancer, diagnostic biomarkers, endogenous control, microRNA, NGS, normalization strategy

## Abstract

**Introduction:**

Cervical cancer causes approximately 350,000 deaths each year. The availability of sensitive and specific diagnostic tests to detect cervical cancer in its early stages is essential to improve survival rates.

**Methods:**

In this study, we compared two strategies for selecting endogenous controls: miRNA profiling by small-RNA sequencing and a commercially available microfluidic card with 30 recommended endogenous controls preloaded by the manufacturer. We used the RefFinder algorithm and coefficient of variation to select endogenous controls. We selected the combination of miR-181a-5p and miR-423-3p as the most optimal normalizer. In the second part of this study, we determined the differential expression (between tumor/non-tumor groups) of microRNA in cervical cancer FFPE tissue samples. We determined the comprehensive miRNA expression profile using small-RNA sequencing technology and verified the results by real-time PCR. We determined the relative expression of selected miRNAs using the 2^-ΔΔCt^ method.

**Results:**

We detected statistically significant upregulation of miR-320a-3p, miR-7704, and downregulation of miR-26a-5p in the tumor group compared to the control group. The combination of these miRNAs may have the potential to be utilized as a diagnostic panel for cervical cancer. Using ROC curve analysis, the proposed panel showed 93.33% specificity and 96.97% sensitivity with AUC = 0.985.

**Conclusions:**

We proposed a combination of miR-181a-5p and miR-423-3p as optimal endogenous control and detected potentially significant miRNAs (miR-320a-3p, miR-7704, miR-26a-5p). After further validation of our results, these miRNAs could be used in a diagnostic panel for cervical cancer.

## 1 Introduction

Cervical cancer is the fourth most commonly diagnosed cancer in women. In 2020, approximately 604,000 new cases were diagnosed, and about 342,000 deaths were recorded worldwide (1, 2). In most cases, the initiating event leading to a malignant overgrowth is persistent infection with human papillomavirus (HPV) and its subsequent integration into the host DNA. Viral oncoproteins are involved in the inactivation of tumor-suppressor genes, inactivation of DNA repair mechanisms, and disruption of physiological apoptosis (3, 4). Prophylactic vaccines against high-risk HPV types are now available and are generally recommended for females and males aged 11-12 years and older where economically feasible. Diagnostic screening for cervical cancer is based on a cytologic examination called a Pap smear (Pap test) and HPV nucleic acid detection (HPV test) (5, 6). The Pap smear has a sensitivity of about 47% and a specificity of about 65%. The Pap smear is highly influenced by the quality and location of the cytology smear, inflammation, or infection of the cervix. A positive HPV test indicates the presence of HPV DNA but does not necessarily indicate the presence of malignancy (sensitivity 88.32% and specificity 54.92%) (7). Both screening tests have limited diagnostic efficacy for cervical cancer, so there is an opportunity to identify and develop new diagnostic methods and markers, for example based on miRNA detection.

miRNAs are small non-coding RNAs that negatively regulate gene expression at the post-transcriptional level. In 2002, the first evidence of microRNA (miRNA) involvement in cancer (B-cell chronic lymphocytic leukemia) was described (8). Later, it was shown that some miRNAs are dysregulated in different types of cancer, making miRNAs potentially promising cancer biomarkers (9). Due to their small size (~22 nucleotides), miRNAs are relatively resistant to degradation, are well detectable in tissues, blood samples, or body fluids, and are easy to extract (10). Despite significant advances in miRNA research, cancer-specific biomarkers that are reproducible across different studies and can be used in clinical practice have not yet been described.

This study discusses the issue of data normalization in miRNA quantification and endogenous control selection by comparing different endogenous control selection strategies. This study also focuses on the identification of significant miRNAs that have the potential to be used in the diagnosis of cervical cancer. Profiling of miRNA expression using advanced small-RNA sequencing technology provides a complete overview of the expression of all miRNAs annotated in miRbase (11). This study aims to detect significantly deregulated miRNAs in the tumor group compared to the control group and to propose a new diagnostic miRNA panel for cervical cancer.

## 2 Materials and methods

### 2.1 Clinical samples

In this study, we analyzed formalin-fixed and paraffin-embedded cervical tissue samples provided from the archives of The Fingerland Department of Pathology, University Hospital Hradec Kralove. The study sample comprised 66 females aged 35-65 years (median; 47 years) who underwent conization (n = 26) or hysterectomy (n = 40) for HPV-associated squamous cell carcinoma (SCC) of the uterine cervix during the period 2010-2020. All cases were reviewed by an experienced gynecopathologist and re-staged according to the both original 2017 version of the 8th TNM classification (12) and the FIGO 2018 staging system (13). Additionally, the presence of HPV DNA was confirmed in all cases by PCR with the subtype provided. The control sample comprised 30 females aged 40-80 years (median; 51 years) who underwent hysterectomy due to uterine leiomyomas or prolapse during the same period. Microscopically, the cervical tissue was devoid of any dysplastic or malignant lesions in all these cases upon review (JL). Ethical approval was obtained from the Ethics Committee of the University Hospital Hradec Kralove, reference number: 202112 P02.

### 2.2 RNA extraction and cDNA synthesis

Three to five 4-5 µm thin slices of formalin-fixed, paraffin-embedded tissue samples were deparaffinized using xylene and 96% ethanol. Total RNA was extracted using a commercial kit according to the manufacturer´s protocol: FFPE RNA/DNA Purification Plus Kit (Norgen Biotek, Thorold, Canada). The extracted RNA was eluted in 30 µl of RNase-free water. The concentration and purity of extracted total RNA were measured on a DS-11 FX Spectrophotometer/Fluorometer (DeNovix Inc., Wilmington, DE, USA) by measuring the optical density at 260 nm and 280 nm, respectively, 260/280 and 260/230 ratios. RNA samples were stored at -80°C until downstream analysis. The cDNA synthesis was performed using the TaqMan™ Advanced miRNA cDNA Synthesis Kit (ThermoFisher Scientific, Waltham, MA, USA) according to the manufacturer´s protocol. The input amount of RNA for reverse transcription was 9 - 10 ng. Amplified cDNA was diluted 10-fold with 0.1M TE buffer.

### 2.3 Small-RNA sequencing

Two pools of extracted RNA from malignant and non-malignant samples were prepared for sequencing analysis. The RealSeq^®^-Biofluids, Plasma/Serum miRNA Library Kit for Illumina^®^ sequencing (RealSeq Biosciences, Santa Cruz, CA, USA) was used for library preparation. The whole procedure was carried out according to the manufacturer’s protocol. The input amount of RNA was 150-170 ng. The concentration and purity of the prepared library were verified using the High Sensitivity DNA Kit on a 2100 Bioanalyzer Instrument (Agilent Technologies, Santa Clara, CA, USA) and the Qubit dsDNA HS Assay Kit on an Invitrogen Qubit 4 Fluorometer (ThermoFisher Scientific). The prepared library was diluted to 4nM with 5% PhiX sequencing Control added to the prepared library and sequenced using MiSeq Reagent Kit v2 1 x 50 on MiSeq System Sequencer (Illumina, San Diego, CA, USA) with 2 million reads per sample. The acquired data were evaluated with miRge3.0, Python package for comprehensive analysis of small RNA sequencing data (14), for microRNA expression using database miRbase and the integrated DESeq2 module for differential expression. FastQC v0.11.9 and MultiQCv1.13 were used for quality control of the sequencing reads. The output sequencing data were further evaluated using the DEApp web interface (15). This interface combines three algorithms for differential expression analysis of edgeR (16), voom (17), and DESeq2 (18).

### 2.4 Quantitative real-time PCR of miRNAs

Initially, real-time PCR analysis was performed using TaqMan™ Advanced miRNA Human Endogenous Control Card (ThermoFisher Scientific) for endogenous control selection, a pre-spotted microfluidic card containing 30 specific assays of potential endogenous controls miRNA. The reaction was performed using two pools of samples (cancer and control) using TaqMan™ Fast Advanced Master Mix (ThermoFisher Scientific) and ABI PRISM 7900HT real-time PCR machine (ThermoFisher Scientific). Primary data were analyzed using the Thermo Fisher Connect Platform (19) (ThermoFisher Scientific). Secondly, real-time PCR was performed on all individual samples to verify the results of the previous analyses. miRNA quantification was performed using TaqMan Fast Advanced Master Mix (ThermoFisher Scientific) with specific TaqMan Advanced miRNA Assays (ThermoFisher Scientific) on Rotor-Gene Q (Qiagen, Hilden, Germany). The following assays were selected as candidate endogenous controls: hsa-miR-451a (478107_mir), hsa-miR-25-3p (477994_mir), hsa-miR-423-5p (478090_mir), hsa-miR-103a-3p (478253_mir), hsa-miR-484 (478308_mir), hsa-let-7i-5p (478375_mir), hsa-miR-181a-5p (477857_mir), hsa-miR-423-3p (478327_mir). As potentially relevant miRNAs were used: hsa-miR-26a-5p (477995_mir), hsa-miR-4286 (478096_mir), hsa-miR-7704 (480576_mir), hsa-miR-4454 (478104_mir), hsa-miR-320a-3p (478594_mir). All reactions were performed in triplicates with a reaction volume of 10 µl (2.5 µl cDNA). During the preparation of the master mix, the ratio of reagents was maintained according to the manufacturer’s protocol. No template control, no reverse transcriptase control, and an inter-run calibrator were included in each run. A pool of all tested samples was used as the inter-run calibrator. PCR reaction conditions followed the manufacturer’s protocol (enzyme activation at 95°C for 20 seconds, followed by 40 cycles: denaturation at 95°C for 3 seconds and annealing at 60°C for 30 seconds). Initial fluorescence data were evaluated using Rotor-Gene Q Series Software (Qiagen). Standard qPCR was used only for validation of endogenous controls (selected by array qPCR card and NGS) and for validation of diagnostic miRNAs (selected by NGS). Relative expression was determined using the 2^-ΔΔCt^ method (20).

### 2.5 Statistical methods and algorithms

The RefFinder algorithm (21), which combines the BestKeeper (22), NormFinder (23), geNorm (24), and Delta Ct (25) algorithms by geometric mean, was used to select the endogenous control. These algorithms are based on calculating the stability value of a given reference gene and then comparing the stability value between endogenous controls: the lower the stability value, the more suitable the endogenous control. BestKeeper is based on the determination of Pearson’s correlation coefficient (r), standard deviation (SD), and coefficient of variation (CV). The NormFinder algorithm uses statistical analysis of variance (ANOVA) to determine the stability of gene expression. GeNorm calculates the stability score M as the average pairwise variability (V) compared to other reference genes. It then ranks all reference genes by M value and sequentially excludes those with the most significant M value. Finally, it combines the most appropriate endogenous genes according to the geometric mean. The Delta Ct method compares the relative expression of two candidate reference genes. If the Delta Ct value remains constant between samples, both genes are stably expressed. If the Delta Ct is not constant, one or both of the selected reference genes are not stably expressed. The stability of expression is determined by comparing all reference genes. The coefficient of variation (CV) was used to determine the variability of selected endogenous controls. The CV value was calculated from the Ct values normalized to the endogenous control (ΔCt) of the control samples.

Statistical analysis was performed using GraphPad Prism 9 (GraphPad Software Inc., San Diego, CA, USA). Data were log transformed to reduce skewness and then tested for normality using the Shapiro-Wilk test. Differential expression was calculated using the 2^-ΔΔCt^ method. Differences in expression between tumor and control groups were compared using Student’s t-test for data fitting a normal distribution and Mann-Whitney U test for non-normal distribution. Multiple logistic regression was used to determine the sensitivity and specificity and to construct the receiver operating characteristic (ROC) curve of the proposed diagnostic panel of selected miRNAs. The level of statistical significance was set at P < 0.05 and significant Fold Change at 2.

## 3 Results

### 3.1 Endogenous control selection

**Figure 1:**
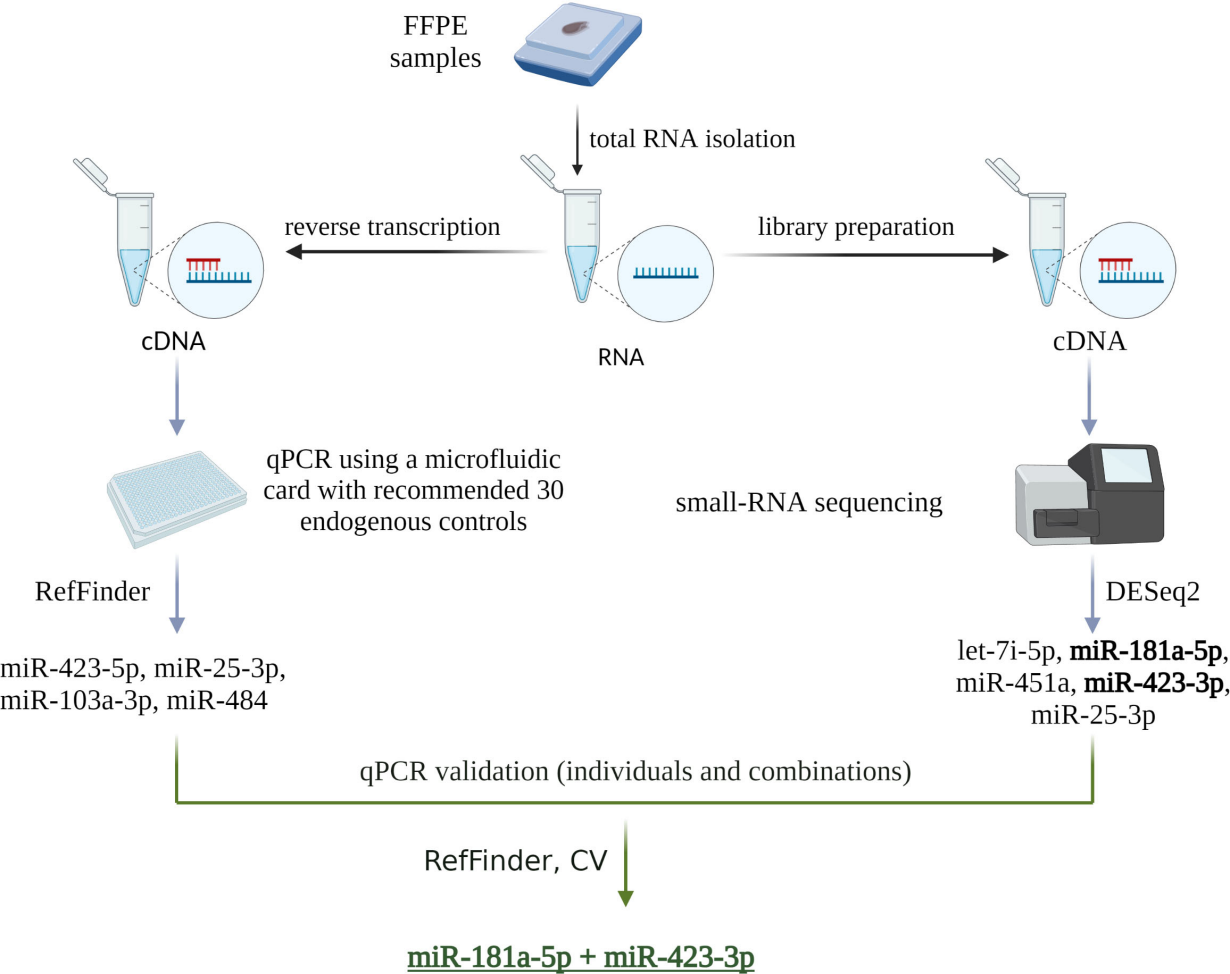
Schematic of the two endogenous control selection strategies used in this study. The first strategy was based on a microfluidic card with 30 endogenous controls pre-selected and recommended by the manufacturer. The second strategy was based on small RNA sequencing analysis. The results of both strategies were then validated by qPCR. Individual miRNAs and their combinations were evaluated using the RefFinder algorithm and by calculating CV, which identified miR-181a-5p and miR-423-3p as the most appropriate endogenous controls. Created with BioRender.com.

**Figure 2:**
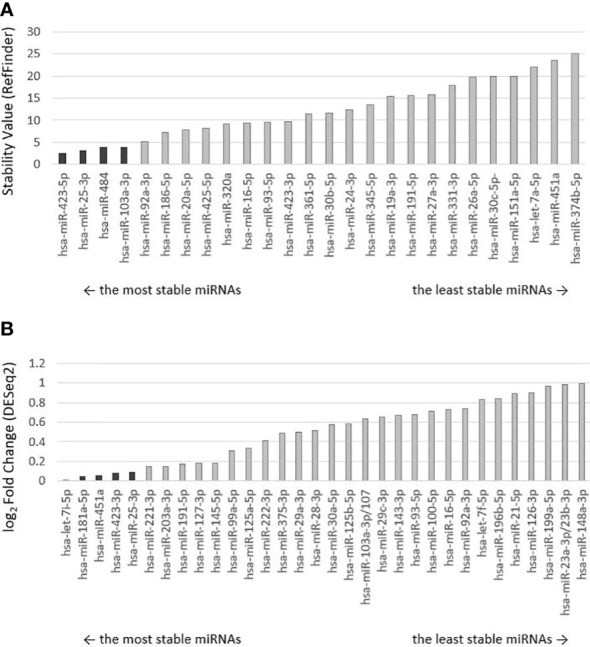
Ranking of miRNA controls by stability. Selected endogenous miRNAs are darkly highlighted. **(A)** The order of endogenous control miRNAs preselected on the microfluidic card by the manufacturer was determined by the RefFinder algorithm, which calculated and assigned a stability value to each miRNA. The lower the stability value, the more stably expressed miRNA and the more suitable it is for use as an endogenous control. **(B)** Ranking of selected endogenous miRNAs from small-RNA sequencing analysis evaluated by DESeq2 algorithm, which performed differential expression analysis. Low-expression miRNAs were excluded from the analysis. miRNAs are ranked from the lowest log2 Fold Change value (the lesser the value, the greater the stability between malignant and non-malignant samples).

Two strategies were used to select the most appropriate endogenous control (**Figure 1**). The first strategy used a reduced selection of 30 recommended endogenous controls from the manufacturer (ThermoFisher Scientific), pre-spotted on a microfluidic card. Four miRNAs were selected as suitable to be used as endogenous control: miR-423-5p, miR-25-3p, miR-103a-3p, and miR-484 based on a stability value <5 as determined by the RefFinder algorithm (**Figure 2A**). The second strategy was based on the results of a small-RNA sequencing analysis. Based on the profiling of all miRNAs, those with an average base value <100 and log2FoldChange >2 were discarded. Five miRNAs with the lowest log2FoldChange were selected as potentially usable: let-7i-5p, miR-181a-5p, miR-451a, miR-423-3p, miR-25-3p (**Figure 2B**).

**Figure 3:**
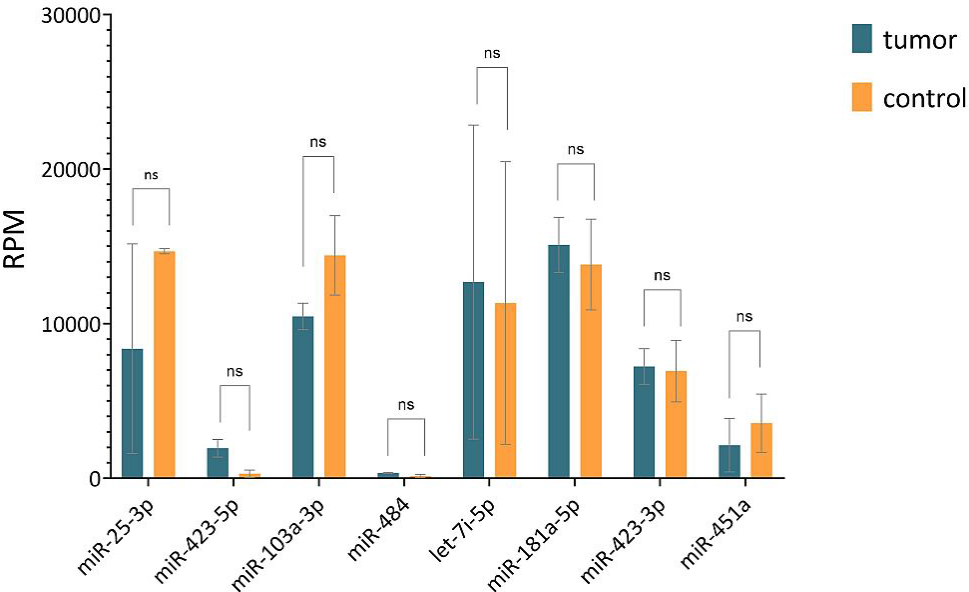
Comparison of RPM values of 8 selected endogenous controls obtained from sequencing analysis of tumor and control groups. Horizontal lines represent median values and error bars indicate 95% confidence intervals. The abbreviation "ns" means not significant difference.

**Figure 4:**
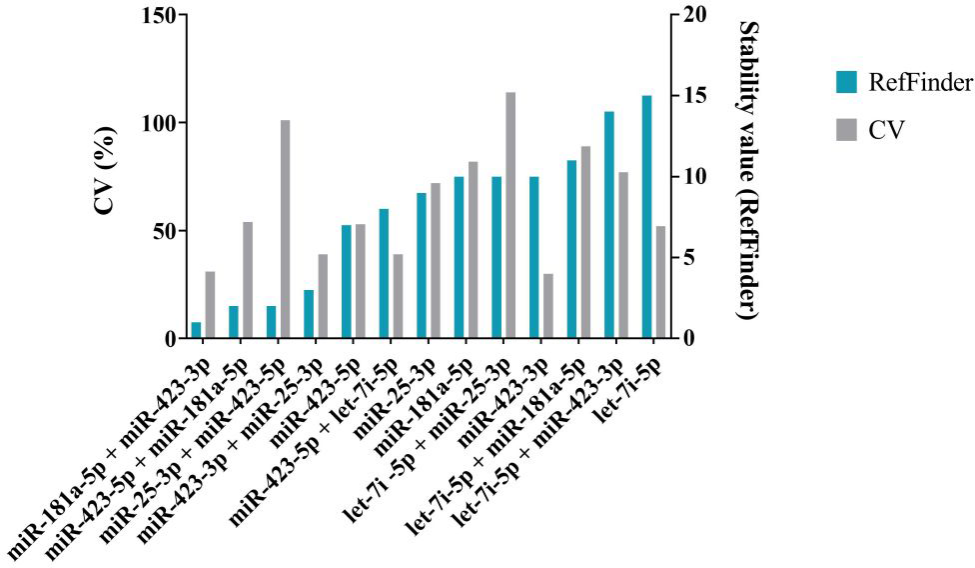
Analysis of a selection of endogenous controls and their combinations. The coefficient of variation (%) is shown in grey on the left y-axis. The stability values of the RefFinder algorithm, shown in blue, are plotted on the right y-axis. The lower the CV and the lower the stability value, the more stable the miRNA is and the more suitable it is for use as an endogenous control. The combination miR-181a-5 + miR-423-3p has the lowest stability value and CV value. It was selected as endogenous control.

The results of the sequencing analysis of eight selected endogenous controls (miR-25-3p identical in both strategies) are shown in **Figure 3** (plotted RPM) for the tumor and control groups. The expression of these eight endogenous control miRNAs was verified by real-time PCR on individual samples from the cohort. During validation, miR-451a, miR-103a-3p, and miR-484 were discarded because expression (Ct > 40) was not detected in all cohort samples. The Ct values obtained in the real-time PCR validation were combined by geometric mean to generate potential combinations of the selected miRNAs. The stability value (RefFinder) and CV were determined (**Figure 4**). The lower the CV and stability value, the more appropriate the endogenous control. The RefFinder algorithm evaluated the combination of miR-181a-5p + miR-423-3p as the most stable endogenous control. According to the CV results, miR-423-3p (CV = 30%) and the combination miR-181a-5p + miR-423-3p (CV = 31%) showed the lowest variability. The combinations miR-181a-5p + miR-25-3p and miR-423-5p + miR-423-3p were discarded due to CV > 200% (high variability). The combination miR-181a-5p + miR-423-3p was evaluated as the most reliable endogenous control according to the RefFinder algorithm and CV calculation. Therefore, it was selected as an endogenous control for further analysis of relative miRNA expression in cervical cancer samples. Both miRNAs selected as endogenous controls were detected by the second strategy (small-RNA sequencing analysis).

### 3.2 Determination of significantly deregulated miRNAs

**Figure 5:**
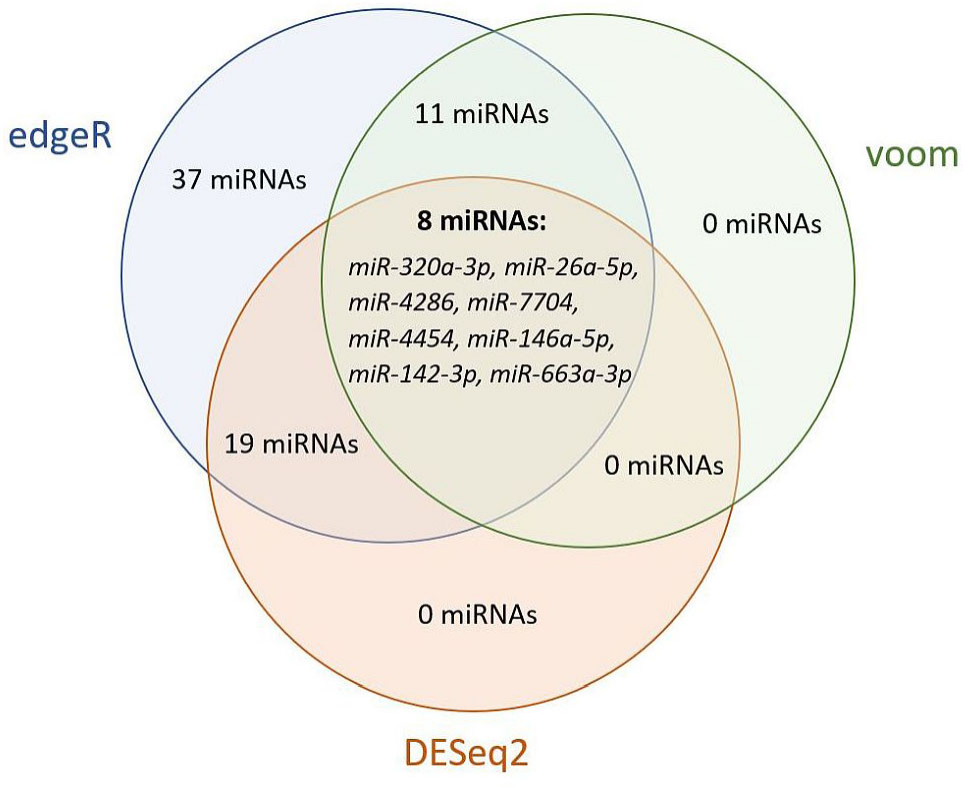
Venn diagram of statistically significant differentially expressed miRNAs by edgeR, voom, and DESeq2 algorithms. Evaluation using the DEApp web interface. The edgeR algorithm identified 75 miRNAs, the voom algorithm identified 19 miRNAs and the last algorithm DESeq2 identified 27 miRNAs that were statistically significantly differentially expressed. The intersection of all three algorithms identified 8 differentially expressed miRNAs.

**Figure 6:**
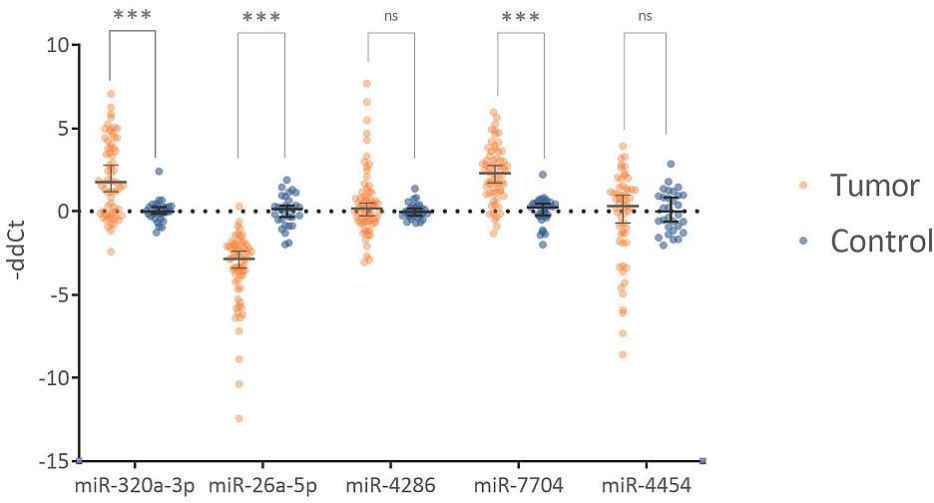
Comparison of relative expression of tested miRNAs. The Fold Change values are plotted on a logarithmic scale. miR-320a-3p and miR-7704 show statistically significant upregulation. miR-26a-5p shows statistically significant downregulation. miR-4286 and miR-4454 are not statistically significant. Horizontal lines represent median values, and error bars show a 95% confidence interval. The abbreviation "ns" indicates not a significant difference, and *** indicates a p-value <0.001.

To determine significantly deregulated miRNAs in cervical cancer, we compared the miRNA expression profile of carcinoma samples with controls. A total of 2730 miRNA isoforms were sequenced, of which 2523 showed no expression and were discarded. The expression of the remaining 207 miRNA isoforms in tumor and control groups are shown in **Supplementary Figure 1**. The output data were analyzed using the DEApp web interface, which includes three algorithms (edgeR, voom, DESeq2). After evaluation of the sequence data, 2523 miRNA isoforms showed no expression and were discarded. The edgeR algorithm identified 75 miRNAs, the voom algorithm identified 19 miRNAs and the last algorithm DESeq2 identified 27 miRNAs that were statistically significantly differentially expressed. The intersection of all three algorithms identified 8 differentially expressed miRNAs: miR-320a-3p, miR-26a-5p, miR-4286, miR-7704, miR-4454, miR-146a-5p, miR-142-3p, miR-663a-3p (**Figure 5**). miRNAs with low expression (base mean <100) were discarded (miR-146a-5p, miR-142-3p, miR-663a-3p). For the remaining five miRNAs, expression was verified by real-time PCR using endogenous control miRNAs selected in the first step of the study. We detected statistically significant upregulation of miR-320a-3p (FC = 4.145; p-value < 0.001), miR-7704 (FC = 4.778; p-value < 0.001), and downregulation of miR-26a-5p (FC = -11.144, p-value < 0.001). In **Figure 6**, we can see the comparison of the relative expression of target miRNAs.

**Figure 7:**
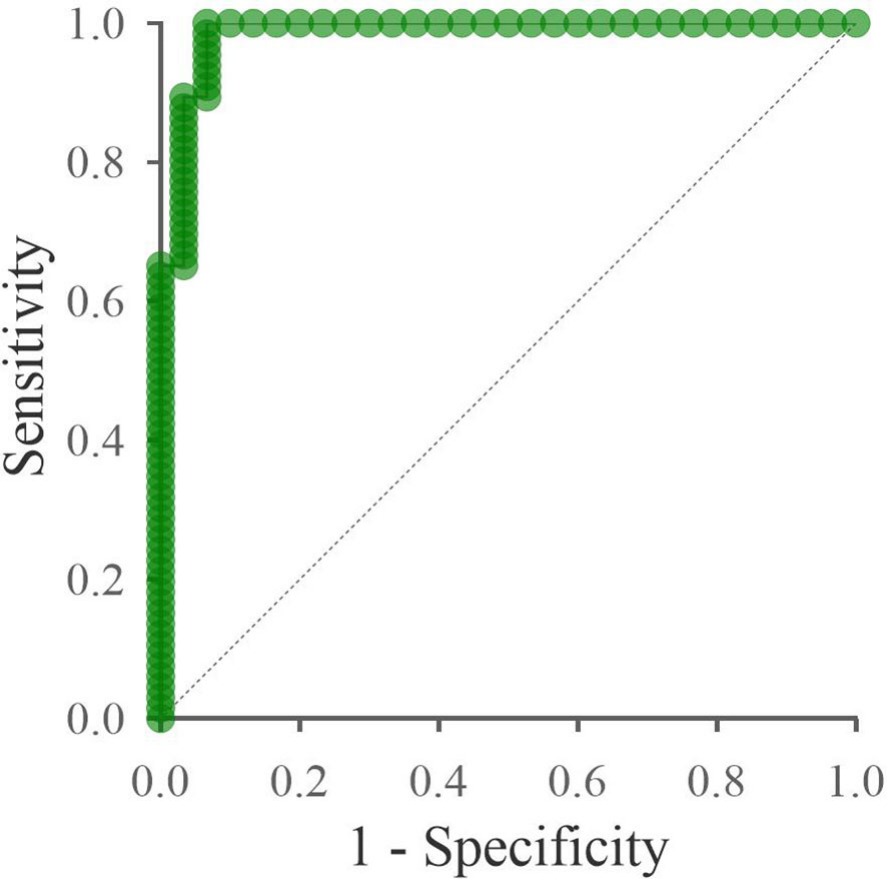
ROC curve of the proposed panel: miR-320a-3p, miR-26a-5p, miR-7704. The x-axis shows the 1-specificity value, and the y-axis shows the sensitivity. The specificity of the proposed panel is 93.33%, and the sensitivity is 96.97%. The area under the ROC curve is 0.985. The dashed line indicates the area under the curve equal to 0.5.

Significantly deregulated miRNAs (miR-320a-3p, miR-26a-5p, miR-7704) could be potentially used as diagnostic biomarkers of cervical cancer. Combining significant miRNAs into a diagnostic panel can increase specificity and sensitivity. In **Figure 7**, we can see the ROC curve showing the sensitivity and specificity of the proposed panel. The specificity of the proposed panel is 93.33%, and the sensitivity is 96.97%. The cutoff value was set at 0.5. The area under the ROC curve is 0.985. *The Predicted vs. Observed* classification table is shown in **Table 1**. The proposed panel detected 2 false positives and 2 false negatives. There were 64 cases correctly diagnosed as positive and 28 cases correctly diagnosed as negative.

**Table 1:** Results of ROC analysis of the three miRNAs combination.

Classification table	*Predicted negative*	*Predicted positive*	Total
*Observed negative*	**28**	**2**	30
*Observed positive*	**2**	**64**	66
Total	30	66	96

## Discussion

In recent years, considerable progress has been made in studying miRNAs, supported by several thousand publications. Nevertheless, miRNA-based markers are advancing very slowly to enter clinical practice. The specificity of individual miRNAs is limited. A single miRNA can target tens to hundreds of mRNA targets, and a single mRNA can bind multiple miRNA molecules. For these reasons, miRNA panel-based diagnostics is the most advanced area of miRNA research. Moreover, a major limitation of miRNA-based research field is the problem of reproducibility studies, which could be caused by improper data normalization. Currently, there are no universally accepted or recommended normalizers (endogenous controls). The endogenous control should have high stability, quantification should be detected in all samples and should be always tested for a specific tissue and project. An endogenous control with variable and unstable expression can lead to misinterpretation of data and false conclusions (26). Very often, the selection of an endogenous control is based on literature sources only but is not further optimized and validated on the samples tested.

The first objective of this study was to select the most appropriate endogenous control for valid normalization of miRNA expression data in our cohort of cervical tissue sample. The combination of miR-181a-5p + miR-423-3p was selected as the most appropriate endogenous control. A similar study was performed by Nilsen et al., who selected the combination of miR-151-5p, miR-152-3p, and miR-423-3p as an endogenous control in their study of hypoxic miRNA markers in cervical cancer (27). Partially similar results were obtained by Babion et al., who used small RNA sequencing to select an endogenous control for their studies of miRNA expression in cervical scrapings (28, 29). They chose miR-423-3p as an endogenous control, but combined it with RNU24 (small nucleolar RNA). Previously, several studies have shown that small nucleolar RNA is not a suitable endogenous control due to variable expression and different lengths (30–32). In contrast, our second selected miR-181a-5p has not yet been described as an endogenous control in studies focusing on miRNA expression and cervical tissue. Further validation studies are needed to confirm its potential as an endogenous control.

In this study, we compared two endogenous control selection approaches, small RNA sequencing, and microfluidic array card, with 30 recommended endogenous controls. Although miRNA profiling by sequencing is more expensive and time-consuming, our results confirmed that it leads to a more appropriate endogenous control, and the selection is not limited to the recommended endogenous controls, which may not be appropriate in all tissue types. Similarly, the study by Kaur et al. discusses the issue of endogenous control selection in determining miRNA expression in bone metabolism (30). They also base their choice of an endogenous control on sequence data and highlight the consequences of an incorrectly selected unstable endogenous control. After evaluating the results of our two approaches, the selected endogenous control should be validated by qPCR, the most widely used method for miRNA quantification to date. This is a critical step because the quantification principles of sequencing and qPCR are different. The same strategy was used by Drobna et al. to study T-ALL (33). Their endogenous control was selected based on a comprehensive analysis of expression stability by miRNA-seq followed by validation by qPCR.

The second aim of this study was to identify important miRNAs that might have the potential for cervical cancer diagnosis in the future. Small RNA sequencing was used to identify differentially expressed miRNAs in the cervical cancer group compared to controls, and potential miRNAs were validated by qPCR. Statistically significant upregulation of miR-320a-3p and miR-7704 and statistically significant downregulation of miR-26a-5p were identified. miR-320a-3p belongs to the miR-320 family, whose role in malignancies is quite complex. Members of the miR-320 family have anti-tumor roles, but their oncogenic effect has also been described in some cancers (34). Specifically, miR-320a-3p has been described as a tumor suppressor in non-small cell lung cancer (35), hepatocellular carcinoma (36), or colon cancer (37). Conversely, it has been described as an oncogenic miRNA in prostate cancer (38) or ovarian cancer (39, 40). In our study, we identified oncogenic activity in cervical cancer. In contrast, some studies have reported anti-tumor activity of miR-320 in cervical cancer (41, 42), Interestingly, they demonstrated the negative regulation of miR-320 on the expression of genes that have important functions in tumor progression, invasion, and angiogenesis. miR-320 could exhibit oncogenic activity during tumor initiation, but conversely, its expression could be reduced during metastasis and angiogenesis, as it has been shown to be associated with suppression of epithelial-mesenchymal transition inhibition (43). However, further research is needed to determine the specific molecular role of miR-320a-3p in cervical cancer carcinogenesis.

A direct association of miR-7704 with cervical cancer has not been described, but a significant upregulation was detected in our study. There are very few studies focusing on miR-7704 and its role in cancer. In a study by Mahlab-Aviv et al., they studied miRNA expression in the spliceosome fraction in breast cancer (44). They described miR-7704 as a tumor suppressor and identified the HAGLR gene as its nuclear target. However, they also pointed out that the patterns of miRNA expression in the spliceosome fraction may differ significantly from those in the cytoplasmic fraction, so these results cannot be properly compared. In contrast, the oncogenic role of miR-7704 in cancer, as identified in our study, was also described by Zheng et al. (45). They investigated the role of chemotherapy resistance in head and neck cancer and found the upregulation of miR-7704 and its anti-tumorigenic effect. Further studies are needed to establish the role of miR-7704 in cancer, especially in cervical cancer. The tumor suppressor activity of miR-26a-5p in cancer has been extensively described in many studies and has great potential in diagnostics (46–48). Rather, some studies have already addressed its therapeutic potential and identified its target mRNA in cervical cancer. A study by Dong et al. demonstrated that miR-26a-5p is a tumor suppressor that inhibits cell proliferation and invasion of cervical cancer cells under physiological conditions by targeting protein tyrosine phosphatase type IVA1 (49). In contrast, Li et al., 2022 identified a different target (50). They demonstrated a negative correlation of miR-26a-5p with hydroxysteroid dehydrogenases like 2 and described a therapeutic potential in increasing miR-26a-5p expression. Although the tumor-suppressive effect of miR-26a-5p in cancer has been proven, knowledge of its therapeutic use remains limited.

Our study used FFPE tissue, which has its own limitations in terms of sample quality, heterogeneity, and invasive sampling. On the other hand, it is a readily available material sufficient for the initial analysis of miRNA expression in malignancies. In addition, a biopsy of the affected tissue is still crucial for cancer diagnosis. However, it is an invasive type of sampling that is almost always limited by the sampling site. Diagnostics based on detecting miRNAs in blood have great potential for clinical practice. The miRNAs released from tumor cells into the blood reflect the complex heterogeneity of the entire tumor lesion and are not limited to the sampling site. The next step in our research is to validate our findings in blood samples, preferably in a larger and different cohort of patients. The number of samples in our study is relatively small and limited and may have influenced our results and conclusions. Independent validation studies are recommended. On the other hand, due to the careful and specific selection of endogenous controls, it is likely that our results are not affected by incorrect data normalization.

In conclusion, this study focused on the issue of data normalization and selection of an endogenous control when determining relative miRNA expression in the cervical tissue. To select an endogenous control, we recommend miRNA profiling (array or small-RNA sequencing) followed by validation by qPCR with data evaluation using the RefFinder algorithm. We selected the combination of miR-181a-5p + miR-423-3p as the most appropriate endogenous control for cervical tissue. In this study, we detected significant upregulation of miR-320a-3p and miR-7704 and downregulation of miR-26a-5p in cervical cancer samples compared to controls. We proposed a potential diagnostic panel of miR-320a-3p, miR-26a-5p, miR-7704, and miR-181a-5 + miR-423-3p (endogenous control) in cervical cancer. The results obtained need to be verified in further independent validation studies.

## Data availability statement

The datasets presented in this study can be found in online repositories. The names of the repository/repositories and accession number(s) can be found below: https://www.ncbi.nlm.nih.gov/geo/, GSE227693.

## Ethics statement

The studies involving human participants were reviewed and approved by Ethics Committee University Hospital Hradec Králové. The patients/participants provided their written informed consent to participate in this study.

## Author contributions

ST, BI, PH contributed to the conception and design of the study, LJ organized and reviewed the database of the patients and sample data, ST, BI, MP, GB, and VH performed the laboratory analyses ST, BI performed the statistical analysis, ST wrote the first draft of the manuscript, LJ, BI, PV, and PH reviewed the manuscript. All authors contributed to the article and approved the submitted version.
