# Supplementary material for: Selection of endogenous control and identification of significant microRNA deregulations in cervical cancer
Source: Front Oncol. 2023 Apr 24;13:1143691. doi: 10.3389/fonc.2023.1143691 (PMC10164982; doi:10.3389/fonc.2023.1143691)
Supplement: Supplementary file 1 [file DataSheet_1.docx]

**
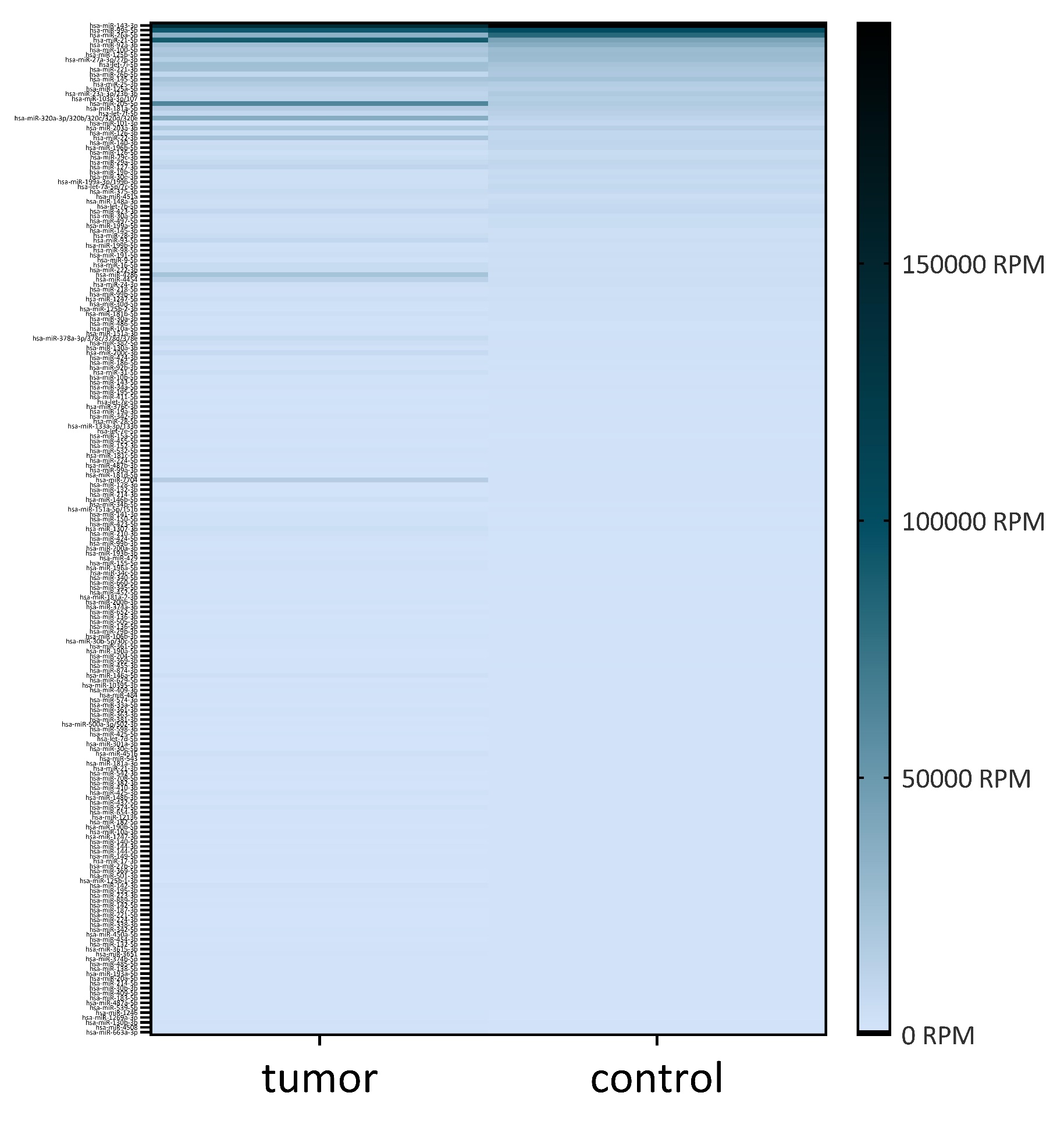
Supplementary Figure 1** Heat map showing miRNA isoforms detected by small RNA sequencing in cervical cancer tumor group and control group. A total of 207 miRNA isoforms with detectable expression (RPM> 100) are plotted. The RPM value is plotted on the right y-axis and the names of individual miRNAs are plotted on the left y-axis.
